# Postharvest senescence of green vegetables and the role of glutathione metabolism: current state of knowledge and future research opportunities

**DOI:** 10.3389/fpls.2026.1918891

**Published:** 2026-09-09

**Authors:** Gale G. Bozzo, Dhanya Sivakumar, Yifan Yan, Alejandra Beltrán Plascencia

**Affiliations:** Department of Plant Agriculture, University of Guelph, Guelph, ON, Canada

**Keywords:** ascorbate, Brassicaceae, glutathione, glutathione disulfide, oxidative stress, postharvest, senescence, vegetable

## Abstract

The rapid progression of senescence contributes to the postharvest perishability of green vegetables including Brassicaceae commodities. Tissue yellowing due to chlorophyll degradation is a hallmark feature of senescing green vegetables and is associated with the accumulation of reactive oxygen species. Moreover, oxidative stress metabolism in vegetables is exacerbated by postharvest temperature abuse and the senescence-related phytohormone ethylene. Glutathione is a primary antioxidant that detoxifies reactive oxygen species in plants. Glutathione status in postharvest vegetables is partially dependent upon the degree to which it is oxidized to glutathione disulfide, as well as whether glutathione is recycled from glutathione disulfide via the ascorbate-glutathione recycling pathway. Apart from reactive oxygen species management, the concentration of glutathione within Brassicaceae commodities is a consequence of its participation in non-antioxidant metabolism such as glucosinolate biosynthesis and protein glutathionylation. Although not well described for postharvest vegetables, glutathione status in postharvest vegetables is postulated to be impacted by catabolism to its constituent amino acids. In the model Brassicaceae plant *Arabidopsis thaliana*, glutathione is catabolized to the metabolic intermediates cysteinylglycine and 5-oxoproline, and finally to cysteine, glycine and glutamate. Improved knowledge of these metabolic events in commercially relevant Brassicaceae commodities could provide novel biochemical targets for offsetting postharvest senescence of their edible vegetables. In addition, we discuss the current state of knowledge on the application of exogenous glutathione prior to postharvest storage as a strategy to lessen the negative impact of oxidative stress mediated deterioration of highly perishable vegetables.

## 1 Introduction

**Figure 1:**
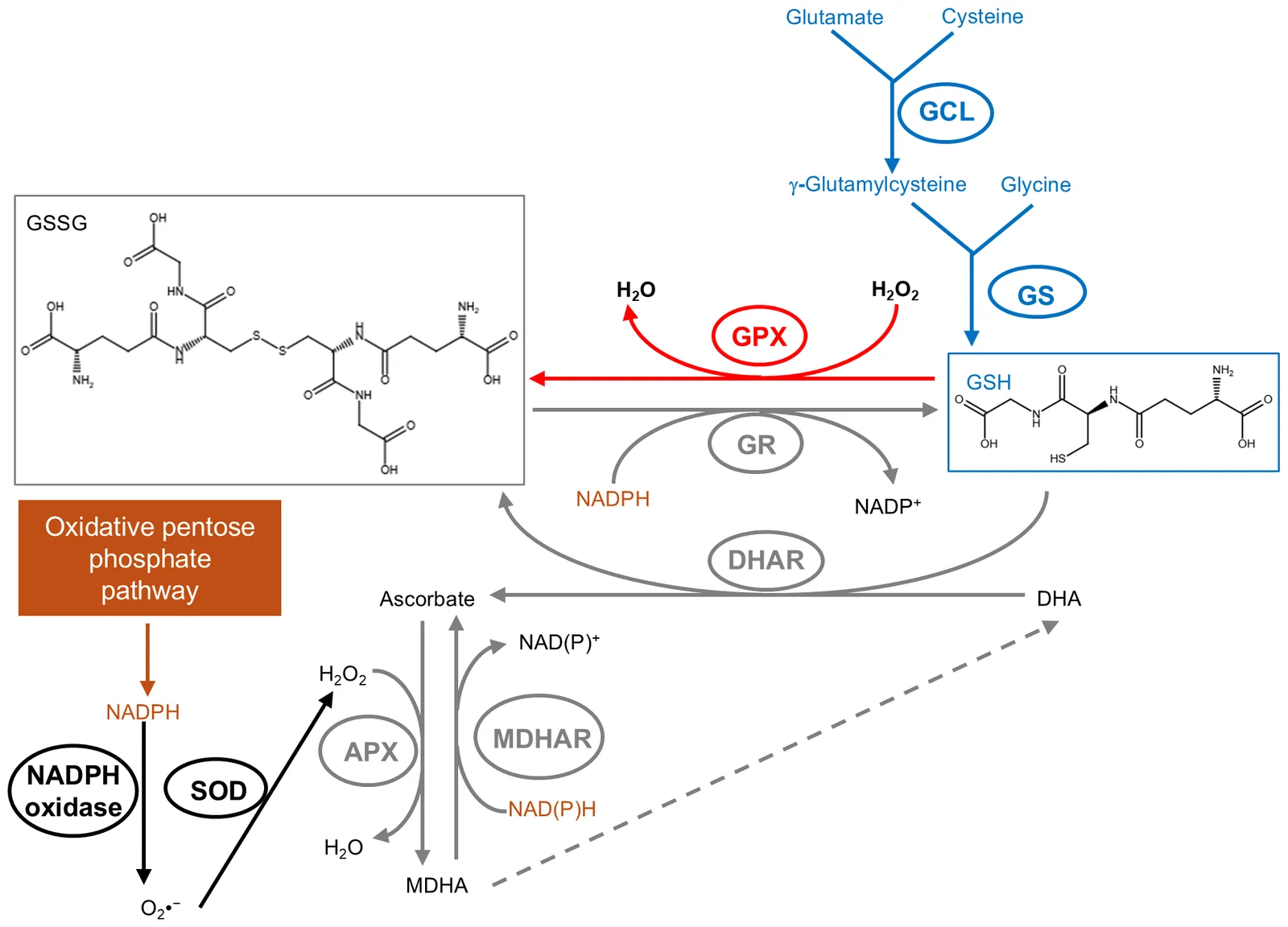
The canonical ascorbate-glutathione (GSH) recycling pathway of plants. GSH biosynthesis reactions are represented by blue arrows. Ascorbate-GSH recycling pathway reactions are represented by gray arrows. Hydrogen peroxide generating enzymes are represented by black arrows. Glutathione peroxidase (GPX) reaction is represented by the red arrow. Other abbreviations include: APX, ascorbate peroxidase; DHA, dehydroascorbate; DHAR, dehydroascorbate reductase: GCL, glutamate-cysteine ligase; GR, glutathione reductase: GS, glutathione synthase: GSSG, glutathione disulfide; MDHA, monodehydroascorbate; MDHAR, monodehydroascorbate reductase; SOD, superoxide dismutase. Glutathione chemical structures were drawn with Chemical Sketch Tool (https://www.rcsb.org/chemical-sketch; accessed on 10 February 2026). Scheme was adapted from Lum et al. (2016) and Aghdam et al. (2020).

Fresh vegetables are dietary sources of the antioxidants ascorbate and glutathione (GSH). This includes commodities of the Brassicaceae family. The primary role of antioxidants within the plant is their participation in the chemical reduction of reactive oxygen species (ROS) to less harmful metabolites, although non-antioxidant roles exist in cruciferous vegetables (e.g., conversion of GSH to glucosinolates). Plant ROS include superoxide (O_2_•^−^), singlet oxygen (^1^O_2_), hydrogen peroxide (H_2_O_2_), and the hydroxyl radical (HO•). Superoxide is derived from oxygen (O_2_) and electrons leaked from electron transport chains in the chloroplasts and mitochondria of plant cells. Superoxide is also generated by a plasma membrane-bound NADPH oxidase (**Figure 1**). Superoxide can be further metabolized to hydrogen peroxide by superoxide dismutase. Both hydrogen peroxide and superoxide can serve as substrates for hydroxyl radical generating reactions, including metal-dependent Fenton reactions (Richards et al., 2015). Singlet oxygen is derived from the triplet excited state of chlorophyll molecules in the thylakoid membranes of plant cell chloroplasts (Triantaphylidès and Havaux, 2009). ROS concentrations fluctuate to varying degrees throughout a plant’s life cycle. ROS regulate various developmental and physiological processes in plants, including those related to floral reproduction, root growth, and leaf stomatal closure (Martin et al., 2022). A buildup of ROS beyond homeostatic levels is classified as oxidative stress, which can promote damage to proteins, DNA, and membranes of plant cells. Moreover, oxidative stress events are associated with the development of senescence symptoms (e.g., yellowing of leaves) and hence loss of quality in postharvest horticultural commodities. ROS management in plants is dependent upon the ready availability of antioxidants including the chemically reduced forms of ascorbate and glutathione, the latter of which is the focus of this review.

In this review the abbreviation GSH refers to the chemically reduced form of glutathione (i.e., γ-glutamyl-cysteinyl-glycine), whereas total glutathione refers to the sum of GSH and its oxidized form glutathione disulfide (GSSG). GSH is a key thiol that occurs in many living organisms, including a high prevalence in edible horticulture. The antioxidant GSH is a biochemical linchpin for oxidative stress management in senescing plants, as well as a precursor for specialized metabolites (e.g., glucosinolates) in Brassicaceae plants. The antioxidant function of GSH is due to a reduced sulfhydryl group within the cysteinyl portion of the molecule (Noctor et al., 2024). ROS-mediated oxidation of the sulfhydryl group within GSH promotes its own dimerization, yielding GSSG. GSH can directly scavenge hydrogen peroxide, superoxide and the hydroxyl radical, but these processes are vastly inefficient relative to enzyme-driven mechanisms (e.g., glutathione peroxidase, GPX) (Jomova et al., 2024). GSSG is recycled back to GSH via NAPDH-dependent glutathione reductase (GR) activity. The GR step is a major component of the ascorbate-GSH recycling pathway (i.e., Foyer-Halliwell-Asada pathway) which occurs in various plant cells. Components of this recycling pathway occur in the mitochondria, plastids, cytoplasm, peroxisomes and apoplast. Another component of the ascorbate-GSH recycling pathway is the regeneration of ascorbate from dehydroascorbate by GSH-dependent dehydroascorbate reductase activity (**Figure 1**). In postharvest tissues the supply of NADPH to power this recycling pathway is due in part to NADP dehydrogenases that operate in the pentose phosphate pathway (e.g., glucose-6-phosphate dehydrogenase) and carbon sources derived from sucrose (Lum et al., 2016; Aghdam et al., 2020). Unfortunately, NAPDH tends to be limited in senescing vegetables due to a decline in the activities of NADPH-generating enzymes in the oxidative pentose phosphate pathway (Wang et al., 2024a). Apart from this recycling pathway, ROS are also detoxified by GPXs, which are GSH-dependent enzymes that convert hydrogen peroxide to water. The capacity of postharvest vegetables to maintain their glutathione and ascorbate pools in a highly reduced state is dependent upon a fully operational Foyer-Halliwell-Asada pathway. In green vegetables postharvest senescence is often associated with the loss of GSH including a portion that is oxidized to GSSG, as well as the partial to complete collapse of the ascorbate-GSH recycling pathway. Various reviews have been published on the role of the ascorbate-GSH recycling pathway and GSH in developing and stressed plants, including factors that impact subcellular compartmentation (Noctor et al., 2012, 2024; Dorion et al., 2021; Foyer and Kunert, 2024). Here, the link between senescence of green vegetables and alterations in GSH metabolism for antioxidant related processes are compared to the current state of knowledge on the impact of exogenous GSH on green vegetables as well as the role of lesser explored GSH processes during vegetable senescence, such as glucosinolate metabolism, *S*-glutathionylation and GSH catabolism.

## 2 Factors driving the postharvest senescence of green vegetables

Green vegetables (e.g., broccoli, kale, lettuce, mustard greens, spinach) are highly perishable with postharvest handling. For example, losses of green vegetables range from 18% to 63% in the United States retail sector (Porat et al., 2018). Vegetable losses in the postharvest supply chain including within commercial distribution centers are due in part to sub-optimal temperature management and humidity control practices that promote senescence and hence decreased shelf-life. Some vegetable commodities (e.g., broccoli, lettuce) require a strict low temperature range (i.e., 0 to 2 °C) to promote optimal shelf-life and quality, whereas others are best preserved at 7 to 10 °C (e.g., cucumber) or require warmer temperatures of 13 to 18 °C (e.g., squash, tomato) to limit chilling-related disorders (Thompson and Kader, 2016). Generally, less than two weeks of shelf-life is typical for most green vegetables and fruits marketed as vegetables (e.g., ripe tomato) when refrigerated at their optimal storage parameters (Sousa-Gallagher et al., 2016). Failure to meet these conditions accelerates senescence, which is associated with leaf discoloration (e.g., yellowing), wilting, decay and electrolyte leakage (Kou et al., 2014; Koukounaras et al., 2007). The accelerated senescence of green vegetables compromises their quality and hence marketability.

Postharvest senescence of horticultural products can be delayed by low temperature but accelerated with temperature abuse (i.e., warmer temperatures than those that are recommended to optimize freshness and high quality). Advanced postharvest senescence is due to a multitude of factors including inefficiencies in the supply chain such as inadequate cold storage facilities for the tight regulation of commodity-specific temperature requirements (Ishangulyyev et al., 2019). Postharvest temperature abuse can occur at multiple points in the supply chain including transportation, distribution centers, and within retail operations (Ndraha et al., 2018). A recent study of the long-distance transport of select fruits (e.g., strawberry) and vegetables (e.g., cucumber) from Spain to Switzerland found that warming events occur along the supply chain, specifically during transport from the packinghouse to the distribution center and thereafter during transport to the retail market (Shoji et al., 2022). With respect to leafy green vegetables, a United States study found that warming events above the acceptable limit of 5 °C were documented for 17% of shipments that were continually monitored from their point of origin (e.g., California) to distribution centers across the country (e.g., Ohio) (Brown et al., 2016). Moreover, a North American study established that the realized temperatures within display cabinets at various grocery stores range from –1 to 19 °C, with coincident relative humidities of 34% to 93% (Nunes et al., 2009). Temperatures within vertically oriented refrigerated display cabinets can be impacted by the depth and height position of the shelving (de Frias et al., 2015; Kou et al., 2015; Xie et al., 2021). Kou et al. (2015) found that bagged spinach (*Spinacia oleracea*) is exposed to warmer temperatures when located at the front of a vertically oriented and open refrigerated display cabinet. Similarly, de Frias et al. (2015) determined that greater decay of bagged spinach occurs at the front relative to the back of vertical open display cases. The exposure of green vegetables to temperature abuse promotes physiological alterations associated with senescence such as yellowing and/or browning, as well as losses of moisture and fresh weight. For example, rapid visual quality loss occurs in wild rocket (*Diplotaxis tenuifolia*) and baby leaf lettuce (*Lactuca sativa*) under the sub-optimal temperature of 10 °C relative to storage at 6 °C (Raffo et al., 2021). Storage under controlled temperature settings is a standard practice within industrialized nations. Conversely, in developing nations the lack of cold chain infrastructure contributes to approximately 82% more food losses than the global average (Jarman et al., 2023). Thus, we need to develop reliable and feasible strategies for distribution and marketing of horticultural products in supply chains, including within jurisdictions that lack adequate cold chain infrastructure.

Postharvest senescence of vegetables is intimately associated with starch and sucrose catabolism and respiration of their derived reducing sugars. Respiration is a metabolically expensive process. In developing plants, diurnal respiration can consume up to 60% of a plant’s carbohydrate budget in order to produce ATP for growth and maintenance processes (Amthor et al., 2019). Unlike preharvest plants where the carbohydrate status is replenished in actively photosynthesizing leaves or translocated to sinks from source tissues, respiration is an all-consuming process in postharvest vegetables given that photosynthesis is limited, and source-sink relations are not in operation. Typically, respiration of green vegetables exceeds that of other produce such as root vegetables. For example, the respiration rate of spinach leaves (e.g., 230 mg CO_2_ kg^–1^ h^–1^) at 20 °C is nearly a magnitude greater than that of carrot and potato (Gross et al., 2016). The high surface area-to-volume ratio of most leafy vegetables contributes to their high respiration rates with postharvest handling and storage (Shezi et al., 2024). The perishability of leafy green vegetables is linked with their low carbohydrate content and the respiratory consumption of simple sugars derived from starch breakdown (Yu et al., 2021, 2022). Interestingly Hasperué et al. (2015) reported a near halving of the concentrations of total sugars, fructose and glucose in broccoli florets after 21 days at 4 °C, which overlapped with chlorophyll degradation. Exogenous sucrose supplied to broccoli effectively limits senescence during storage at 10 °C for 7 days (Ghimire et al., 2023). Harvest timing also impacts soluble sugars levels in green vegetables. Kale leaves harvested in the afternoon contain more total sugars than plants harvested in the morning, the latter of which displayed more senescence symptoms after 6 days of postharvest storage at 20 °C (Casajús et al., 2021). Similarly, slightly less senescence is evident in broccoli harvested at the end of the day than heads picked in the early morning; the latter contained less starch at harvest and less soluble sugars after storage at 20 °C (Hasperué et al., 2011).

Respiration is exacerbated in vegetables exposed to temperature abuse. Hertog et al. (1998) used flow through respirometry-based assessments of O_2_ consumption and CO_2_ evolution to establish that aerobic respiration in endive leaves is more pronounced at warm temperatures (i.e., between 16 to 28 °C) relative to cooler temperatures. Similarly, Spadafora et al. (2016) reported a near doubling of the respiration rate in packaged wild rocket leaves when assessed immediately following their removal from storage at 10 °C as opposed to those sampled from 0 °C. A recent investigation of the highly perishable toon bud identified a much earlier global induction in the expression of aerobic respiration enzyme genes during storage at 20 °C than at 4 °C; the vegetables stored at 20 °C also accumulated more tricarboxylic acid pathway intermediates (e.g., malate) (Zhao et al., 2023).

The yellowing of green vegetables is a visible sign of the degradation of chlorophylls that occurs with postharvest senescence. Postharvest yellowing and chlorophyll loss occur in broccoli (*Brassica oleracea* var. *italica*, Ahlawat et al., 2022), cabbage (*Brassica oleracea* var. *capitata*, Kramchote et al., 2012), cucumber (*Cucumis sativus*, Schouten et al., 2002), kale (*Brassica oleracea* var. *sabellica*, Casajús et al., 2021), green leaf lettuce (Martínez-Ispizua et al., 2022), pak choi (*Brassica rapa* var. *chinensis*, also known as pak choy, Able et al., 2005), arugula (*Eruca sativa*; Koukounaras et al., 2006), and spinach (Bergquist et al., 2006). Temperature abuse can accelerate senescence-related yellowing. For example, arugula leaves undergo more leaf yellowing at suboptimal temperatures (i.e., ≥ 5 °C) than at 0 °C (Koukounaras et al., 2007). In addition, enhanced yellowing and/or off-odor formation with temperature abuse truncates the shelf-life period of spinach (Kou et al., 2014), broccoli (Yan and Bozzo, 2026), baby lettuce, and wild rocket leaves (Raffo et al., 2021; Sivakumar and Bozzo, 2024).

Chlorophylls are degraded to colorless tetrapyrroles. Most chlorophyll catabolism steps occur between the chloroplast thylakoid membrane and chloroplast envelope (Hörtensteiner, 2013). Our current understanding of chlorophyll catabolism is based on research performed with the model Brassicaceae plant *Arabidopsis thaliana*. The leaves of *A. thaliana* and other *Arabidopsis* spp. are likely edible, but these plants are not commercially relevant. Nonetheless, information on biochemical mechanisms associated with leaf senescence in *A. thaliana* provides foundational knowledge for comparative mechanisms in its botanical relatives. In soil-bound *A. thaliana* plants the senescence-related reduction in leaf chlorophyll concentration precedes the dismantling of chloroplasts (i.e., reduction in chloroplast size and the disappearance of starch granules) and the degradation of the photosynthetic machinery (e.g., light harvesting complex I and II proteins) (Tamary et al., 2019). Leaf chloroplasts transform into plastoglobuli-rich gerontoplasts during on-the-plant senescence (Golczyk et al., 2014). Similar ultrastructural alterations occur in various lettuces during postharvest storage (Wagstaff et al., 2007). Protease containing vesicles occur in senescing broccoli florets (Bárcena et al., 2020). These proteolytic vesicles contain the cysteine protease SAG12 (encoded by *SENESCENCE ASSOCIATED GENE 12*) which degrades plastidial proteins and is a well-known marker of leaf senescence (Aloryi et al., 2023). A greater and more advanced accumulation of *SAG12* transcripts and those of the related *SAG13* occur in a NAD-accumulating mutant of *A. thaliana* (i.e., *onset of leaf death5*), which senesces earlier than wild-type plants (Schippers et al., 2008).

Postharvest senescence is regulated by a gene transcription program that is distinct from that of plants undergoing natural senescence (Großkinsky et al., 2018; Ghimire et al., 2023). Nonetheless downstream biochemical processes such as chlorophyll and antioxidant losses are evident for both types of senescence. Increased expression of senescence-related genes occurs in kale, cabbage, and broccoli during storage at 25 °C (Ahlawat and Liu, 2021). These transcriptional alterations overlap with the upregulated expression of chlorophyll catabolism and ethylene biosynthesis/signaling genes. Interestingly, chlorophyll depletion in broccoli heads is exacerbated by the phytohormone ethylene (Gómez-Lobato et al., 2014). Warm storage temperatures promote ethylene-related senescence. For example, pak choi yields more ethylene and senesces more readily at 20 °C than at 2 °C (Able et al., 2005). Interestingly, Li et al. (2022) determined that broccoli florets produce less ethylene and remain greener when fumigated with the senescence inhibitor diacetyl prior to 25 °C storage. Many Brassicaceae vegetables are sensitive to the negative effects of exogenous ethylene (e.g., atmospheric concentrations as low as 10 nL L^–1^), including when they are proximal to ethylene-emitting horticulture within a commercial refrigeration unit (Cocetta and Natalini, 2022).

Ethylene negatively impacts antioxidant metabolism within vegetables. The loss of ascorbate in spinach leaves is accelerated following their immersion in ethephon, a plant-growth regulator that releases ethylene (Gergoff et al., 2010). Similarly, Hodges and Forney (2000) established that exogenous ethylene exacerbates the loss of total ascorbate and total glutathione in spinach leaves stored at 10 °C. The ethylene antagonist 1-methylcyclopropene suppresses senescence and the loss of antioxidants. 1-Methylcyclopropene lessens the proportion of ascorbate loss in cauliflower and broccoli after 4 days at 20 °C (Ma et al., 2010, 2011). Yuan et al. (2010) determined that 1-methylcyclopropene-treated broccoli heads had 67% more ascorbate in their florets and a longer shelf-life at 20 °C than untreated heads. To a similar extent, 1-methylcyclopropene limits the degree of ascorbate loss in Chinese kale (*Brassica alboglabra* cv. ‘Nairecutiao’) stored at 20 °C for 7 days (Sun et al., 2012). 1-Methylcyclopropene effectively suppresses the degree of senescence in spinach leaves and limits the loss of ascorbate and GSH (Grozeff et al., 2010). Moreover, ethylene could also be critical for GSH degradation in other horticulture including fruits, given that 1-methylcyclopropene delays the loss of GSH in ‘AC Harrow Crisp’ European pear (*Pyrus communis*) fruit during prolonged refrigeration at 0 °C (Flaherty et al., 2018). Interestingly the loss of ascorbate during dark-induced senescence is unaffected in *A. thaliana* ethylene-signaling mutants (Hamada et al., 2025), implying that ethylene does not regulate ascorbate degradation. The same study found no change in the dynamics of ascorbate loss during prolonged dark incubation of various ascorbate-GSH recycling pathway mutants. Conversely, there is crosstalk between glutathione redox metabolism and ethylene in developing plants. Riyazuddin et al. (2022) established that ethylene production is heightened in plants of an *A. thaliana* mutant containing a T-DNA insertion in *GPX5* (i.e., *At*3G63080) relative to wild-type plants. The potential for crosstalk mechanisms between GSH metabolism and ethylene production and signaling have not been well explored in postharvest horticulture.

## 3 Glutathione and oxidative stress management in postharvest vegetables

A common feature of plant senescence is the accumulation of ROS beyond homeostatic levels, which promotes organellar and cellular disorganization. ROS concentrations tend to be elevated with senescence-promoting conditions (e.g., abuse of temperature) that induce yellowing in many green vegetables, including broccoli (Li et al., 2022; Zhang et al., 2022; Yan and Bozzo, 2026), pak choi (Song et al., 2023), and Chinese flowering cabbage (*Brassica rapa* subsp. *parachinensis*; Li et al., 2021a). ROS are mainly chloroplastic and peroxisomal in origin within the photosynthetic tissues of developing plants, whereas the mitochondrial electron transport pathway (specifically complexes I, II and III) is primarily involved in generating ROS in non-photosynthetic tissues, although some contribution from other cellular compartments can occur (Huang et al., 2016; Decros et al., 2019). In illuminated leaves, superoxide is formed in the chloroplast electron transport chain from the incomplete reduction of O_2_, and singlet oxygen is derived from the transfer of energy to O_2_ by photosystem II chlorophylls that are excited to their triplet state (Mayta et al., 2019). Light exposure does not appear to be a strict requirement for chloroplast-derived ROS metabolism. Increased superoxide and hydrogen peroxide levels occur within plastoglobuli-accumulating chloroplasts of pak choi leaves stored at 20 °C under darkness (Song et al., 2020). ROS accumulation coincides with the decomposition of mitochondria and chloroplasts in packaged Chinese flowering cabbage stored at 20 °C (Mou et al., 2023). To date, there is no definitive conclusion as to the respective contribution of chloroplasts and mitochondria to ROS accumulation in postharvest vegetables. It is likely that chloroplast breakdown is a major contributor to cellular ROS production. Leaf senescence is associated with increased expression of genes encoding chlorophyll catabolism and ROS biosynthesis enzymes (e.g., respiratory burst oxidase homolog) (Fan et al., 2018), as well as the senescence marker *SAG12* (Zhang et al., 2011; Bresson et al., 2018). Additional research is required to better understand the subcellular source(s) of ROS in postharvest vegetables. Genetically encoded biosensors fused to subcellular targeting peptides (e.g., reduction-oxidation sensitive green fluorescent protein 2 conjugated to oxidant receptor peroxidase 1, a hydrogen peroxide biosensor) are used for the non-invasive fluorescence-based detection of organellar ROS pools *in planta* (Arnaud et al., 2023). Similarly, glutathione biosensors (e.g., glutaredoxin 1 conjugated to a reduction-oxidation sensitive green fluorescent protein) are available to study the impact of oxidative stress on glutathione redox status of organelles in plant cells (Arnaud et al., 2023). ROS biosensor technology represents a feasible diagnostic strategy to diagnose the impact of the various organellar ROS and glutathione pools on the senescence of postharvest vegetables.

### 3.1 Glutathione biosynthesis and derived pathways in postharvest Brassicaceae commodities

In plants oxidative stress-related cell damage is minimized when ROS are directly detoxified by antioxidants (e.g., GSH) or by enzymes (e.g., superoxide dismutase, catalase), including antioxidant-dependent mechanisms (e.g., GPX). With respect to food for human consumption, GSH is ubiquitous in fresh meat, mushroom, and plant products, albeit at varying concentrations (Jones et al., 1992). Based on a strict synopsis of recent publications, GSH concentrations in green vegetables range from 0.05 to 1.85 µmol g fresh weight^–1^ and are an order of magnitude less abundant than order of magnitude less abundant than ascorbate (Mori et al., 2009; Grozeff et al., 2013; Tan et al., 2020; An et al., 2022; Song et al., 2023; Sivakumar and Bozzo, 2024; Yan and Bozzo, 2026). This trend is also apparent in fruits commercially marketed as vegetables. For example, GSH levels are two orders of magnitude lower than ascorbate in the fruit of various types of *Capsicum* peppers (Palma et al., 2020). It is worth noting that ascorbate is the primary antioxidant in plants and is a well-known vitamin that is absent in mammals. Horticultural crops including leafy green vegetables are a rich source of ascorbate (Nurzyńska-Wierdak, 2025). The maintenance of ascorbate status in postharvest vegetables is a major factor in determining the freshness of horticultural produce (Lee and Kader, 2000). Ascorbate status within vegetables is highly dependent upon the availability of GSH and the operation of the ascorbate-GSH recycling pathway, which includes the recycling of GSSG to GSH. GSH status in vegetables is also dependent upon metabolic processes associated with biosynthesis, and dismantling of the thiol to its precursor amino acids.

GSH biosynthesis is initiated with the conjugation of glutamate to cysteine by a chloroplastic glutamate-cysteine ligase to form γ-glutamylcysteine. GSH synthase conjugates γ-glutamylcysteine to glycine yielding GSH. Cytoplasmic and chloroplastic forms of GSH synthase exist in plants. Lim et al. (2014) determined that chloroplastic and cytoplasmic GSH synthesis is involved in plant growth and the response to oxidative stress. GSH and GSSG are distributed throughout the plant cell. GSH is synthesized in the cytoplasm and chloroplast, but also accumulates in peroxisomes, nuclei and mitochondria (Koffler et al., 2013). Export of GSH from the plastid occurs through a chloroquine resistance-like transporter whereas uptake into the mitochondrion requires an ATP-binding cassette transporter (Noctor et al., 2024). Although there is little GSH in the vacuole and apoplast, GSSG predominates in these subcellular spaces, and glutathione conjugates of xenobiotics reside in the vacuole (Noctor et al., 2012).

GSH is distributed throughout the leaf blade (Koffler et al., 2013). GSH concentrations are diurnally regulated in photosynthetically active leaves. For example, leaf GSH pools transiently decline in *Brassica napus* plants following the transition from photoperiodic light to darkness (Hornbacher et al., 2019). Overall, GSH concentrations are little altered with leaf maturation (Koffler et al., 2013), but are affected by abiotic and biotic stresses (Dorion et al., 2021). GSH accumulation is greater in *A. thaliana* leaves cultivated under high light intensity (i.e., 500 µmol m^−2^ s^−1^) relative to plants cultivated under 50% less brightness (Gasperl et al., 2021). At the subcellular level, a spike in GSH levels occurs in chloroplasts and most other cellular compartments (except for vacuoles) within 2 hours of the exposure of *A. thaliana* plants to light (Zechmann, 2017).

Postharvest exposure to light can impact GSH metabolism in stored vegetables. A near 50% increase in GSH levels is described for spinach leaves exposed to periodic (i.e., every 2 or 6 hours) 15-minute pulses of low intensity light (i.e., 30 µmol m^−2^ s^−1^) for 3 days as opposed to untreated leaves (Grozeff et al., 2013). Moreover, Guo et al. (2025) found approximately 50% more GSH in pak choi illuminated with low intensity red light emitting diodes (i.e., 6.5 µmol m^−2^ s^−1^) relative to non-illuminated leaves during 15 °C storage. Light quality also impacts antioxidant-related metabolism and hence shelf-life of leaves. Low intensity blue light (i.e., 10–30 µmol m^−2^ s^−1^) extends shelf-life of amaranth leaves at 4 °C (Jin et al., 2021), which coincides with a more intact ascorbate-GSH recycling pathway, including stable GR. The precise mechanisms driving light-related preservation of GSH in postharvest vegetables remain unknown, but the possibility remains that this is linked to improved maintenance of chloroplast integrity. This is based on evidence that daily exposure of postharvest broccoli to white light or red light for 2 hours limits chlorophyll loss, the degradation of light harvesting complex I and II proteins and photosystem I subunit A protein and delays the appearance of senescence associated vacuoles involved in the dismantling of chloroplasts (Bárcena et al., 2020). As a portion of the GSH biosynthesis and the ascorbate-GSH recycling pathway reside in the chloroplast, it is likely that postharvest lighting maintains chloroplast integrity, and hence limits proteolysis of these GSH-related enzymes.

There is some debate as to whether *de novo* GSH biosynthesis occurs in postharvest horticulture. The arrest of GSH biosynthesis is implied by the unchanged abundance of GSH synthase and glutamate-cysteine ligase gene transcripts in cassava (*Manihot esculenta*) root during storage at 28 °C (Vanderschuren et al., 2014). With respect to Brassicaceae vegetables, Bell et al. (2020) determined that the expression of several glutathione biosynthesis genes are elevated in arugula leaves on day 7 of storage at 4 °C. Similarly, glutamate-cysteine ligase gene transcripts accumulate in chive stored at 3 °C for 12 days (Dai and Yu, 2022). Unfortunately, both the arugula and chive studies did not assess whether GSH pools were altered with the postharvest storage period investigated therein. Recently, Zhang et al. (2025b) established that the glutathione redox status of broccoli transiently increased over 4 days at 15 °C, and this overlapped with the increased gene expression of glutamate-cysteine ligase and GSH synthase. Thus, the possibility remains that *de novo* GSH production occurs in postharvest green vegetables. It remains to be determined whether these phenomena are influenced by the availability of light during storage and/or marketing.

### 3.2 Non-antioxidant GSH metabolism in postharvest Brassicaceae commodities

Apart from oxidation to GSSG, the free GSH pools in plants are also a consequence of the extent to which GSH is conjugated to proteins, xenobiotics and/or metabolized to glucosinolates and phytochelatins. GSH-*S*-transferases conjugate GSH to xenobiotics (e.g., herbicides) as a strategy to limit their negative consequences in the plant (Micic et al., 2024). The conjugation of GSH to proteins occurs under some abiotic and biotic stresses in a process known as *S*-glutathionylation which limits protein damage due to ROS (Dorion et al., 2021). ROS can oxidize cysteine residues in some proteins to sulfenic acid; further oxidation renders sulfinic acid and subsequently the irreversible product sulfonic acid. Some proteins undergo *S*-glutathionylation as a preventative measure against ROS-induced intramolecular disulfide bond formation. *S*-glutathionylation targets include oxidative-stress regulated proteins, such as various glycolysis and tricarboxylic acid cycle enzymes (Dumont and Rivoal, 2019). The activity of the fermentation enzyme alcohol dehydrogenase decreases in the presence of hydrogen peroxide, a treatment that promotes the formation of intramolecular disulfide bridges, but this same enzyme and its activity can be protected by *S*-glutathionylation (Dumont et al., 2018). Various thiol peroxidases such as the pea chloroplastic 2-cysteine peroxidase are glutathionylated (Calderón et al., 2017). *S*-glutathionylation also appears to protect an *A. thaliana* dehydroascorbate reductase from oxidative modification (Waszczak et al., 2014). Although there is little evidence of protein S-glutathionylation in postharvest horticultural products, there is some information on the transcriptional regulation of GSH-*S*-transferases. The expression of eight separate GSH *S*-transferase genes are variably impacted in broccoli during storage at 20 °C (Zhang et al., 2023). Given that many ROS management enzymes (e.g., dehydroascorbate reductase) are susceptible to oxidative modification, additional studies are required to determine whether losses or stable activities of these enzymes in postharvest vegetables coincide with oxidative modification and *S*-glutathionylation, respectively.

Brassicaceae vegetables are a rich source of glucosinolates, a category of thioglucosides. Over 100 different glucosinolates are known, including various aliphatic, aromatic and indole glucosinolates that are distinguished by whether their side chain contains sulfur, and their cursory amino acid(s) (Wu et al., 2021). These glucosinolate biosynthesis pathways generate various thiohydroximate intermediates following conjugation of aldoximes to GSH (Mikkelsen et al., 2004; Ishida et al., 2014). Much of our current knowledge of glucosinolate metabolism in plants and its biological relevance stems from research on the model organism *A. thaliana*. The first evidence that GSH is involved in glucosinolate biosynthesis is that glucosinolates are less abundant in *A. thaliana pad2-1*, a glutamate-cysteine ligase mutant that is GSH-deficient (Schlaeppi et al., 2008). In addition, glucosinolate production is reduced in pak choi following treatment with the GSH biosynthesis inhibitor buthionine sulfoximine (Zhu et al., 2024). Glucosinolate concentrations are an order of magnitude greater than GSH concentrations in *A. thaliana* and pak choi (Schlaeppi et al., 2008; Huseby et al., 2013; Zhu et al., 2024). A screen of 80 broccoli genotypes determined that the glucosinolate content of florets ranged from approximately 0.47 to 57 µmol g dry weight^–1^ (Li et al., 2021c). In developing plants, fully expanded leaves have greater proportions of glucosinolates than young leaves (Brown et al., 2003). By comparison, Shelton (2005) demonstrated that more glucosinolates occur in young radish (*Raphanus sativus*) leaves as opposed to mature leaves of greenhouse-grown plants. Glucosinolates are not evenly distributed throughout the leaf. For example, MALDI-TOF MS imaging and HPLC analysis revealed that nearly 100% more glucoraphanin occurs in the midvein of *A. thaliana* than other parts of the leaf, whereas glucobrassicin is most pronounced in the outer periphery of the leaf blade (Shroff et al., 2008). To a somewhat similar extent, glucosinolate concentrations are greatest in the bottom and white portion of kimchi cabbage leaves that are situated between the central portion and outer leaves of the head (Rhee et al., 2020). Glucosinolate status in *A. thaliana* leaves is under circadian control as concentrations peak during the photoperiod (Goodspeed et al., 2013). Similarly, the concentrations of various aliphatic and indole glucosinolates tend to be highest in broccoli heads harvested near mid-day as opposed to early morning or evening harvests, whereas others (e.g., glucoraphanin) tended to decline throughout the day (Casajús et al., 2020).

Glucosinolate composition of cruciferous vegetables is impacted by postharvest handling. Glucosinolate losses in baby mustard are more evident with storage at 20 °C than at 4 °C (Sun et al., 2020). At 20 °C broccoli head senescence coincides with an approximate 80% loss of the total glucosinolate pool (Casajús et al., 2023). Similarly, major losses of aliphatic glucosinolates and indole glucosinolates occur in broccoli heads packed with ice and held under simulated transport and shelf-life temperatures (i.e., 22–25 °C) (Han et al., 2024).

Breeding and/or biotechnological approaches aimed at elevating GSH content in Brassicaceae vegetables should consider alternate strategies to those targeting glucosinolate metabolism. Unwanted and potentially deleterious consequences are likely in vegetables that are engineered to restrict the accumulation of glucosinolates (e.g., glucosinolate biosynthesis mutation) as a strategy to boost GSH pools for antioxidation. It is worth noting that glucosinolates are critical anti-herbivore compounds. Upon wounding by chewing insects, glucosinolates are catabolized by myrosinases to sulfated aglucones which are then converted to isothiocyanates, pungent compounds that deter further feeding and hence widespread damage to aerial tissues (Blažević et al., 2020). To date it is known that leaf damage by the insect *Mamestra brassicae* is more prevalent in aliphatic glucosinolate-deficient mutants of *A. thaliana* than wild-type plants (Beekwilder et al., 2008). Thus, boosting GSH accumulation in Brassicaceae vegetables at the expense of glucosinolate biosynthesis would likely increase their susceptibility to insect pest damage, which may necessitate the increased application of pesticides during cultivation.

### 3.3 Alterations of glutathione status and the collapse of the ascorbate-GSH recycling pathway in postharvest vegetables

Leaf GSH concentrations tend to decline with natural senescence, including within leaves of soil-bound plants (Vanacker et al., 2006; Ding et al., 2016). This includes a more than 90% loss of GSH in *B. napus* leaves with natural senescence (Bieker et al., 2012). With respect to postharvest handling, temperature is a key factor that influences whether GSH is lost, remains stable or transiently accumulates during the storage period. Many studies have determined that a 50% or more loss of the GSH pool occurs in vegetables under temperature abuse conditions that accelerate the onset of senescence (**Table 1**). Conversely, there is also evidence that GSH pools are minimally altered in some postharvest vegetables. Temperature-specific alterations of GSH profiles are well-established for broccoli inflorescences. More than 50% loss of GSH is evident in broccoli held at 20 °C for up to 6 days (Mori et al., 2009; Raseetha et al., 2013). Comparable losses have been described for broccoli stored at 15 °C (**Table 1**). Conversely, Yan and Bozzo (2026) determined that 62% to 95% spikes in GSH levels occur in broccoli stored at 10 °C, including heads that were partially submerged under a solution of 20 µM ergothioneine (a potent antioxidant derived from mushrooms) prior to storage. Moreover, Yang et al. (2023) determined that GSH concentrations increased by approximately 50% in polyethylene-packaged broccoli held at 4 °C for 25 days, whereas greater losses of ascorbate were apparent over this same time. There is also evidence for similar phenomena in non-Brassicaceae green vegetables. GSH accumulates in spinach stored at 10 °C, which coincided with increased superoxide dismutase and catalase activities and decreased ascorbate concentrations (Hodges et al., 2001).

**Table 1:** Postharvest senescence related alterations in ascorbate and glutathione metabolism signatures in green vegetables.

Commodity	Postharvest treatment and/or storage parameters	Antioxidant-metabolism alterations during postharvest storage	Reference
Broccoli			
cv ‘Erude’	- Whole heads in polyethylene film bags stored at 3 °C or 15 °C for up to 7 days	- 15 °C: > 90% ascorbate* loss; ~70% GSH loss; APX, DHAR and GR activities decreased within 4 days - 3 °C: ~20% ascorbate* loss and stable GSH levels; APX and DHAR activities increased and GR activity stable	Yamauchi and Kusabe (2001)
cv ‘Erude’	- Whole heads in polyethylene film bags treated without (control) or with hot air (50 °C) for 2 hours, then stored at 15 °C for 6 days	- ~85% loss of ascorbate* by day 2 of storage; complete loss of DHA** and ~40% decline of GSH and GSSG by day 6 in control; DHAR, GR and chloroplastic APX activities decreased in control - 1.7-fold more ascorbate in heat-treated broccoli by day 2; DHA** declined ~20%; GSSG levels similar to control; more stable chloroplastic APX, DHAR and GR activities	Shigenaga et al. (2005)
***	- Branchlets packed in polyethylene bag and treated with 0 (control) or 40 nmol ethanol L^–1^ atmosphere - Storage at 20 °C for 5 days	- Nearly complete loss of ascorbate*, ~70% loss of DHA** and GSH in control treatment; ~1/3 decrease of GSSG pool; reduced APX, DHAR and GR activities, and rise in MDHAR activity - Ethanol preserved ~1/3 and ~1/2 of the ascorbate and GSH pools, respectively; smaller DHA and GSSG losses than the control; all ascorbate-GSH recycling pathway enzyme activities stable	Mori et al. (2009)
cv ‘Imperial’	- Broccoli heads partially submerged in 0 (control), 20, 100, or 500 µM EGT and stored at 10 °C for 10 days	- Ascorbate declined 33–46% after day 3; DHA unchanged in the 20 µM EGT treatment but ~50% loss in other treatments. - GSH spiked in control and 20 µM EGT treatment by day 3; GSH dropped ~50% in the other EGT treatments; ~40% GSSG loss after day 3 in all treatments - GR activity decreased and APX increased, with smallest changes in 20 µM EGT treatment; DHAR activity decreased with storage	Yan and Bozzo (2026)
Chinese flowering cabbage			
***	- Immersed in solution of 0 (control) or 100 µM melatonin - Wrapped in polyethylene film bags and stored at 15 °C (80% relative humidity) for 7 days	- Ascorbate declined ~60%; DHA increased 1.5-fold in control and 60% with melatonin; GSH stable in control, ~40% transient increase by day 5 with melatonin; 1-fold more GSSG accumulation in control relative to melatonin treatment; APX, GR, and MDHAR gene expression and activities and DHAR activity greater with melatonin	Tan et al. (2020)
***	- Packaged in polyethylene film (control) or in MAP, and held at 20 °C for 9 days - MAP cabbage exposed to 10 µmol m⁻² s⁻¹ red: blue light emitting diodes (12 h each day)	- Control: ~50% ascorbate* loss; ~20% GSH loss; MAP/light treatment lost 2/3 less ascorbate*, but had ~60% more GSH by day 9; GSSG and DHA** increased ~40% and ~100% respectively in control, but stable in the MAP/light treatment - MAP/light treatment prevented decrease of APX, DHAR, GR and MDHAR activities	Du et al. (2025)
Pak choi			
cv ‘Degao 508’	- Treated with 0 (control) or 5 μL L^–1^ 1-MCP for 12 hours and then stored at 20 °C for 5 days	- Ascorbate and GSH decreased ~70–100% by day 5, regardless of treatment - DHA and GSSG decreased ~20% to 40% - Increase in APX activity greatest with 1-MCP	Song et al. (2020)
cv ‘Jinpin’	- Treated with 1 g L^–1^ taxifolin or control (4% ethanol), then air-dried at 20 °C for 2 hours - Packed in perforated polyethylene bags and stored at 20 °C for 4 days	- Transient ascorbate* loss on day 2 of storage - DHA decreased ~55%, regardless of treatment - GSH spiked ~10% by day 2 (control) and day 4 in taxifolin treatment - GSSG increased ~35% (control) and ~40% (taxifolin) - Taxifolin limited the decline in APX, DHAR and GR activities	An et al. (2022)
***	- Sprayed with 0 (control) or 500 μM melatonin, packed in polypropylene bag (high density), and held at 20 °C for 7 days	- Control: ascorbate* decreased ~45%; DHA** increased ~20%; GSH decreased ~75%; GSSG increased ~75% - Melatonin limited ascorbate* and GSH losses and increases in DHA** and GSSG	Song et al. (2023)
cv ‘Shanghaiqing’	- Pak choi dipped in distilled water for 15 minutes - Stored for 8 days at 15 °C under darkness (control) or 6.5 µmol m^−2^ s^−1^ red light emitting diodes (12 h light/12 h dark cycle)	- Ascorbate* declined ~80% (control) and ~50% (red-light treatment); DHA declined ~50% (control) and ~1/3 (red light) - Spike of GSH by day 2, but decreased ~30–50% thereafter; GSSG increased ~1.5-fold (control), but marginally altered by red-light - APX, DHAR and GR activities decreased and MDHAR activity rose; all activities were greater with red-light	Guo et al. (2025)
Rocket			
cv ‘Letizia’	- Leaves dipped in water (control), 100 μM EGT or 500 μM GSH for 30 min prior to storage - All dip treatments were stored at 4 °C for 17 days or 10 °C for 10 days	- GSH stable; GSSG increased 87% to 140% (control); GSSG stable in GSH-treated leaves - Ascorbate declined ~60% but more rapid at 10 °C; DHA decreased as much as 91% by day 7 at 4 °C (4.5-fold increase thereafter); as much as 73% DHA lost by day 7 at 10 °C	Sivakumar and Bozzo (2024)
Spinach			
cv ‘Vienna’	- Packed in perforated plastic bags and stored at 10 °C for 35 days under ambient air, or ambient air with added ethylene, or controlled atmosphere (10% CO_2_ and 0.8% O_2_)	- Total ascorbate decreased 62%, 83% and 55% under ambient air, with ethylene, and CA, respectively - Total glutathione spiked 82% after 21 days (ambient air), but complete loss with ethylene, and 56% loss under CA - Decreased APX activity largest with ethylene	Hodges and Forney (2000)
cv ‘Bison’	- Treated with 0 (control), 0.1, or 1 µL L^–1^ 1-MCP for 6 h at 23 °C - Packed in low density polypropylene and stored at 23 °C for 6 days	- Control: 94% and 65% loss of ascorbate* and GSH, respectively - 1-MCP (1 µL L^–1^): 80% ascorbate* and 35% GSH losses, and limited accumulation of DHA** and GSSG	Grozeff et al. (2013)

*expressed as ascorbic acid and **as dehydroascorbic acid in publication; cv, cultivar; ***no cultivar listed.

APX, ascorbate peroxidase; CA, controlled atmosphere; DHA, dehydroascorbate; DHAR, dehydroascorbate reductase; EGT, ergothioneine; GR, glutathione reductase; GSH, reduced glutathione, GSSG, glutathione disulfide; MAP, modified atmosphere packaging; 1-MCP, 1-methylcyclopropene; MDHAR, monodehydroascorbate reductase.

There does not appear to be a strong consensus that temperature alone impacts GSH metabolism in pak choi, although like broccoli no single study has simultaneously evaluated the effect of different temperatures on GSH metabolism. Song et al. (2020, 2023) reported 70% or more losses of GSH in senescing pak choi held at 20 °C for up to 7 days. Other studies on this vegetable have reported minor spikes in GSH within 2 days of storage at 20 °C under darkness (An et al., 2022) but ~50% GSH loss and doubling of GSSG are evident with prolonged storage (Guo et al., 2025). Minor and major respective accumulations of GSH and GSSG are evident in senescing Chinese flowering cabbage stored at 15 °C (Tan et al., 2020). This same study established that the plant hormone melatonin delays senescence of Chinese flowering cabbage, which was associated with a transient increase of GSH due to the induced gene expression and activities of ascorbate-GSH recycling pathway enzymes. The application of exogenous melatonin also limits senescence and the degree of GSH loss in pak choi (Song et al., 2023). This absence of a strict relationship between storage temperature and GSH alterations could be a consequence of the brief storage periods used in the studies, which limit the opportunity to observe the full suite of senescence-related metabolic alterations that would occur with prolonged storage. For example, GSH concentrations are relatively unchanged in green bell peppers stored at 4 °C for 15 days, but major losses occur thereafter (Yao et al., 2021).

GSH alterations in postharvest horticulture tend to coincide with a decline in endogenous ascorbate concentrations (**Table 1**). In some commodities such as arugula and spinach, depletion of all the available ascorbate and to some extent the dehydroascorbate pool occurs with storage at warm temperatures (e.g., 15 °C) (Nunes et al., 2013; Cocetta et al., 2014; Amodio et al., 2015). Conversely, less ascorbate degradation is apparent in cruciferous vegetables such as arugula and cabbage with low temperature storage (e.g., 1 to 5 °C) (Hounsome et al., 2009; Kramchote et al., 2012; Amodio et al., 2015; **Table 1**). It is worth noting that the timing and magnitude of ascorbate and/or GSH alterations vary with commodity and their precise storage environment. For example, approximately 75% ascorbate degradation occurs in curly kale after 3 weeks at 1 °C (Hagen et al., 2009). In some cases, these losses are lessened with complementary control strategies such as modified atmosphere packaging and inhibition of ethylene action in the plant (i.e., application of 1-methylcyclopropene) (Raffo et al., 2022; **Table 1**). Nearly equivalent losses of GSH and ascorbate have been described for pak choi at 15 °C to 20 °C, although some fluctuations are evident over the first two to three days of the storage period (Song et al., 2020; Guo et al., 2025). There is evidence that ascorbate degradation in senescing vegetables can occur in the absence of a shift in GSH profiles. Sivakumar and Bozzo (2024) determined that baby wild rocket leaves lose more than 50% of the original ascorbate with storage, although this occurred more rapidly at 10 °C than at 4 °C. Similarly, Grozeff et al. (2013) report an 80% drop in ascorbate concentration and transient accumulation of hydrogen peroxide in bagged spinach leaves held at 23 °C for 3 days followed by 7 days at 4 °C, whereas GSH and GSSG profiles are unchanged. Ascorbate and GSH loss in broccoli tend to be more prominent under warm storage temperatures (**Table 1**). This includes evidence for a drop in the concentration of both the oxidized and reduced forms of both antioxidants, implying their catabolism. The degradation of ascorbate and dehydroascorbate to oxalate and threonate occurs in various salad leaves, including several Amaranthaceae, Asteraceae and Brassicaceae commodities (Dewhirst et al., 2017). The catabolic fates of GSH and GSSG are explored in section 3.4.

The timing of senescence-related events appears to be associated with a progressive shift to a more oxidized glutathione (and ascorbate) redox status. Any losses of GSH that occur with postharvest senescence vegetables overlap to some extent with increased GSSG concentrations (**Table 1**). The increased oxidation of GSH with senescence is a built-in mechanism to trap ROS directly or via the ascorbate-GSH recycling pathway. Across all vegetables, there is no finite and universal GSH: GSSG ratio that is typical of senesced tissues. Nonetheless, it is widely accepted that a transformation of this ratio to a more oxidized state precedes senescence. In okra (*Abelmoschus esculentus*), a drop in the GSH: GSSG ratio to below 1 occurs with the progression of senescence (Li et al., 2023). Grozeff et al. (2010) determined that about half of the chlorophyll content disappeared in spinach leaves held at 23 °C for 6 days which overlapped with a drop of the GSH: GSSG ratio from ~2.5 to ~0.5. Moreover, Tan et al. (2020) found that although GSH was unchanged in Chinese flowering cabbage leaves during storage at 15 °C, there was an increase in GSSG and hence a halving of the glutathione redox potential which overlapped with ROS accumulation. Similarly, a recent study by our group (Sivakumar and Bozzo, 2024) determined that GSSG concentrations more than doubled in baby wild rocket leaves after 17 days at 4 °C. This metabolic alteration was more rapid at 10 °C where senescence was apparent by day 10 of storage, whereas GSH concentrations were unaffected by storage at either temperature.

The loss of ascorbate and GSH in stored vegetables tends to coincide with a diminished efficiency of one or more of the canonical enzymes of the ascorbate-GSH recycling pathway (**Table 1**). Ascorbate losses occur in step with the decreased gene expression for several ascorbate-GSH recycling pathway enzymes including monodehydroascorbate reductase and dehydroascorbate reductase as documented in broccoli, okra and spinach (Hodges and Forney, 2003; Ma et al., 2014; Li et al., 2023). Warm storage temperatures negatively influence the operation of this antioxidant pathway. In fact, repression of the ascorbate-GSH recycling pathway enzymes including monodehydroascorbate reductase and GR limit the recycling of ascorbate in tomato stored at high temperature (Massot et al., 2013). In fresh-cut baby spinach leaves, the collapse of the antioxidant machinery at 20 °C coincides with a greater reduction of the activities of GR, monodehydroascorbate reductase and dehydroascorbate reductase relative to leaves held at 4 °C (Cocetta et al., 2014). Moreover, Ripoll et al. (2019) noted decreased *APX* expression in butterhead lettuce leaves after 1 week at 6 °C. Apart from APX, enzyme-driven reduction and peroxidation of glutathione metabolites is also critical for the efficient operation of the ascorbate-GSH pathway and concomitant sequestration of ROS. APX activity decreases in broccoli, Chinese flowering cabbage, pak choi and spinach stored at warm temperatures between 10 to 20 °C (**Table 1**). Similar trends are also apparent for other ascorbate-GSH recycling enzymes such as GR. Various technologies are available to curtail or limit the decreased activity of ascorbate-GSH recycling enzymes. These include the pre-storage-application of ethanol, 1-methylcyclopropene, melatonin or taxifolin as well as when green vegetables are wrapped in modified atmosphere packaging or exposed to red light during storage (**Table 1**).

GR is a key step in the regeneration of GSH from GSSG to support the efficient operation of the ascorbate-GSH pathway. Typically, most plants contain a minimum of two separate GRs (Trivedi et al., 2013). *A. thaliana* contains two GRs, the cytoplasmic GR1 and two separate GR2 isoforms (Rouhier et al., 2006). GR1 is crucial to maintain glutathione redox status in developing plants, given that lower GSH: GSSG ratios are apparent in *atgr1* mutants (Mhamdi et al., 2010). GSH redox balance is maintained in the chloroplast by GR2 which is dually localized to this organelle and the mitochondrion (Marty et al., 2019). This same study determined that GSH oxidation in the mitochondrial matrix is balanced by the mitochondrial-localized GR2 in cooperation with the GSSG transporter ATM3, an ATP-binding cassette transporter, and the mitochondrial thioredoxin system. It is plausible that GR is pivotal for the maintenance of GSH pools and management of ROS in postharvest senescing leaves. This is based on an accelerated developmental senescence in *A. thaliana GR2* RNAi lines containing 40% to 50% of the GSH recycling activity apparent in wild-type plants, which coincided with more hydrogen peroxide and GSSG concentrations by comparison (Ding et al., 2016).

GSH depletion in postharvest tissues is also a consequence of its limited regeneration by GR. GSH losses coincide with decreased GR activity in pak choi and broccoli during storage at 15 °C and 23 °C, respectively (Raseetha et al., 2013; Guo et al., 2025). Moreover, a reduction in GR activity occurs in broccoli heads with prolonged storage at 10 °C (Wang et al., 2020). Similarly, GR and dehydroascorbate reductase activities decreased by as much as 30% in senescing broccoli florets after 10 days at 10 °C (Yan and Bozzo, 2026). Comparable phenomena are described for broccoli head branchlets and fresh-cut broccoli florets when stored at warm temperatures (Mori et al., 2009; Jiao et al., 2025). These phenomena are not strictly associated with photosynthetic organs as similar trends occur in lotus (*Nelumbo nucifera*) roots (Ali et al., 2023). Apart from temperature, the operation of the ascorbate-GSH pathway is also impacted by the presence of ethylene in the storage atmosphere. For example, the depletion of GSH recycling activity in spinach stored at 10 °C is most pronounced in the presence of exogenous ethylene (Hodges and Forney, 2000).

GPX is an additional GSH-based mechanism for ROS detoxification. Multi-gene GPX families occur in plants. There are 8 GPXs in *A. thaliana*, including those that are localized to one of the plastid, cytoplasm, mitochondrion, plasma membrane or secretory pathway (Attacha et al., 2017). Interestingly, transcriptional regulation of GPX genes seems to be associated with *cis*-regulatory elements in their respective promoter regions, including those that are responsive to low temperature and light (Li et al., 2021b). This implies that induction of GPX gene expression is possible at cold postharvest temperatures. Conversely, Ghimire et al. (2023) found minimal expression of two GPX genes (i.e., *GPX5* and *GPX7*) in broccoli inflorescences during postharvest senescence at 25 °C. Extractable GPX activity is lessened in postharvest vegetables, including those that undergo deterioration with senescence. Wang et al. (2023) established that low GSH levels in discolored cherry radish coincide with lower GPX activity relative to non-discolored radishes following storage under senescence-inducing conditions. A decline in GPX activity overlaps with the postharvest physiological deterioration of cassava root slices (Vanderschuren et al., 2014). Interestingly, this same study found less deterioration in cassava roots overexpressing an *A. thaliana* cytoplasmic GPX (i.e., *AtGPX2*, *At*2G31570.1). Additional studies are required to assess the relative importance of GPX and enzymes of the ascorbate-GSH pathway for oxidative stress management of postharvest green vegetables. This could be achieved by the assessment of the impact of gene ablation and/or overexpression of one or more members of this pathway on antioxidant and ROS profiles in postharvest vegetables. It would be interesting to investigate whether bioengineered vegetables or elite breeding lines containing a fully operational ascorbate-GSH pathway under senescence yield greater GSH concentrations, and an enhanced capacity to mitigate oxidative stress during postharvest handling. This seems plausible given the application of exogenous GSH primes vegetables to maintain their postharvest quality and freshness (as described in section 3.5).

### 3.4 Glutathione status in postharvest vegetables – the case for a focus on catabolism

**Figure 2:**
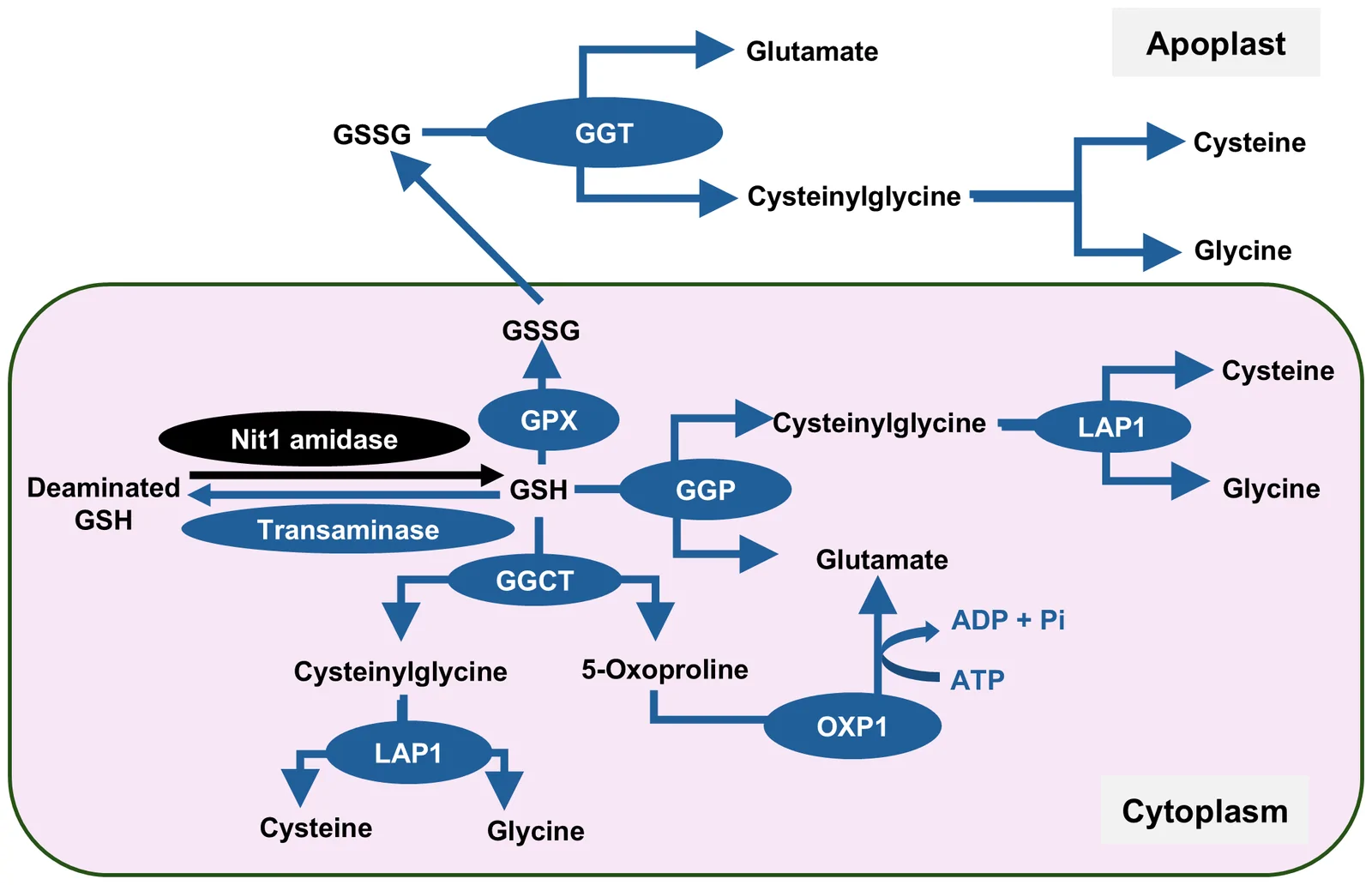
Proposed glutathione catabolism pathway in postharvest vegetables. Apoplastic catabolism steps are represented in the non-shaded region of the scheme. Reduced glutathione (GSH) and glutathione disulfide (GSSG) catabolism reactions are represented by the blue arrows. GSH recycling reaction is represented by the black arrow. GGCT, γ-glutamyl cyclotransferase; GGP, γ-glutamyl transferase; GGT, γ-glutamyl transferase; GPX, glutathione peroxidase; GSH, reduced glutathione; GSSG, glutathione disulfide; LAP 1; leucine aminopeptidase 1; Nit1, nitrilase 1; OXP1, 5-oxoprolinase 1; Scheme was adapted from Peracchi et al., 2017); Niehaus et al. (2019); Ito and Ohkama-Ohtsu (2023).

Although not widely explored in postharvest horticulture, GSH status may also be due in part to GSH and/or GSSG degradation to cysteine, glutamate, and glycine. Approximately 75% of the GSH pool is degraded in *A. thaliana* plants within 20 hours of treatment with the GSH biosynthesis inhibitor buthionine sulfoximine (Ohkama-Ohtsu et al., 2008). Given that a decrease in the efficiency of the ascorbate-GSH recycling pathway can occur with postharvest storage of vegetables, we postulate that the remnant GSH and GSSG pools are susceptible to degradation to their lower molecular weight catabolites (**Figure 2**). The foundation for this possibility is based upon the well-described GSH catabolism pathway in the model plant *A. thaliana*. Specifically, cytoplasmic pools of GSH are converted to 5-oxoproline and cysteinylglycine by γ-glutamyl cyclotransferases or glutamate and cysteinylglycine by γ-glutamyl peptidases (Ito and Ohkama-Ohtsu, 2023). 5-Oxoproline (i.e., pyroglutamic acid) is a cyclized form of glutamate that is converted to glutamate by 5-oxoprolinase (OXP), a cytoplasmic ATP-dependent step (Ohkama-Ohtsu et al., 2008). In *A. thaliana* cysteinylglycine is degraded to glycine and cysteine by leucine aminopeptidase 1 (LAP1, Kumar et al., 2015). Finally, GSSG turnover by γ-glutamyl transferase (GGT) occurs in the apoplast; of the four known GGTs in *A. thalian*a GGT1 and GGT2 are involved in GSSG turnover (Ito and Ohkama-Ohtsu, 2023).

Although there is relatively little information on the relationship between GSH and/or GSSG degradation and postharvest senescence of leafy green vegetables, it is likely that one or more steps of GSH catabolism occur given the following precedents. A proteomics analysis revealed OXP1 levels increase in green bell pepper fruit when held at 4 °C for 16 hours (Xu et al., 2023). Moreover, increased protein abundance of an OXP occurs in the peel of ripening banana (*Musa* spp. AAA group, Cavendish subgroup) fruit (Du et al., 2016). Metabolite profiling also provides evidence of glutathione catabolism in senescent/spoiled produce. A buildup of 5-oxoproline occurs in avocados after 30 days at 5 °C (Pedreschi et al., 2022), and in cold-stored sweet cherry and tomato fruits (Ponce et al., 2021; Liu et al., 2023). Warm storage temperatures promote glutathione catabolism. 5-Oxoproline is more prominent in garlic bulbs held at 22 °C for 290 days relative to storage at 0 °C (Liu et al., 2024), and in spoiled *Volvariella volvacea* mushrooms after 2 days at 25 °C (Xu et al., 2026). Pedreschi et al. (2009) established that pyroglutamic acid is more prominent in the flesh of ‘Conference’ pears that undergo browning during controlled atmosphere storage. Conversely, cysteinylglycine rather than 5-oxoproline accumulates in Chinese cabbage that develop a larger incidence of black spot when packaged with polyvinyl chloride relative to less disordered cabbage that was packaged with oriented polypropylene (Zhang et al., 2025a). Additionally, ripening durian (*Durio zibethinus*) fruit contains a 63 kDa LAP (*Dz*LAP1) that utilizes cysteinylglycine as a substrate (Panpetch and Sirikantaramas, 2021). There is a possibility that similar GSH and GSSG turnover processes occur in senescing Brassicaceae vegetables. LAP families occur within plants. For example, there are 2 and 5 LAP genes in *Capsicum* pepper and tomato, respectively (Muñoz-Vargas et al., 2024). Some LAPs tend to have broad substrate specificity (Tu et al., 2003). Thus, it is likely that most plant LAPs are not involved in GSH catabolism. Transcriptomic and biochemical characterization of cysteinylglycine-utilizing activities in vegetable extracts and/or their recombinant LAP enzymes following expression in heterologous systems are required to determine whether one or more of the putative Brassicaceae OXP1 proteins is involved in GSH degradation during postharvest senescence.

Lastly, GSH losses in postharvest vegetables could be due to their deamination by a transaminase, which is yet to be identified. It is the amino group on the terminal glutamyl region of GSH that undergoes transamination to form deaminated GSH (Peracchi et al., 2017). In the absence of information on the biological relevance of the deaminated GSH, it is considered a damaged metabolite that can be repaired by one or more amidases. Deaminated GSH accumulates in *nitrilase-like protein 1* (*Nit1*) knockouts of *A. thaliana* (Niehaus et al., 2019). Nit1 is a ω-amidase. Nit1 enzymes are distributed across *Escherichia coli*, yeast, mouse and *A. thaliana* (Peracchi et al., 2017; Niehaus et al., 2019). *At*Nit1 is co-targeted to the plastid and cytoplasm (Niehaus et al., 2019). It is proposed that the amidase activity of Nit1 forms α-ketoglutarate and cysteinylglycine from deaminated GSH. To date, it is unknown whether deamination of GSH occurs in senescing plants, including in response to postharvest handling. High expression of *Nit2* occurs in postharvest arugula leaves, although the authors speculated this encodes a nitrilase involved in the conversion of indole-3-acetonitrile to the phytohormone indole-3-acetic acid (Bell et al., 2020). Similarly, increased abundance of transcripts for three nitrilase genes (i.e., *NIT2-1*, *NIT2-2*, *NIT2-3*) was described for broccoli heads after 1 day at 20 °C (Cheng et al., 2023). It is tempting to speculate that one of these nitrilases may be involved in the metabolism of deaminated GSH in postharvest vegetables. Given the precedent for the disappearance of major portions of the total glutathione with postharvest senescence of vegetables, the possibility remains that transamination of GSH, and subsequent repair occur to some extent in postharvest vegetables.

### 3.5 Limiting postharvest senescence in vegetables by boosting glutathione status with exogenous antioxidants

Horticultural products treated with exogenous antioxidants tend to senesce slowly, resulting in a longer shelf-life. In practice, this could be advantageous for vegetables that are washed after harvest to remove field-borne pathogens prior to packaging. These practices often compromise the endogenous levels of ascorbate within some leafy green vegetables, such as watercress and spinach leaves, as compared to non-washed leaves following storage at 4 °C for 10 days (Dewhirst et al., 2017). Washing with chlorinated water has deleterious consequences for the ascorbate content of leafy green vegetables (Raffo and Paoletti, 2022). Similarly, postharvest decontamination of sliced bell peppers with sodium hypochlorite promotes as much as an 80% loss of ascorbate during the soaking process (Ogawa et al., 2018). Dewhirst et al. (2017) postulated that ascorbate losses that occur within salad leaves washed with hypochlorite are likely due to oxidation by chlorine. Approximately 30% more ascorbate degradation is evident in fresh-cut white cabbage following decontamination with sodium hypochlorite or peroxyacetic acid relative to water-rinsed leaves (Vandekinderen et al., 2009). Given the hydrophilic nature of ascorbate its losses in washed vegetables may be due in part to leaching into decontamination and washing fluids. Thus, amending washing solutions with antioxidants could be a viable approach to limiting losses of endogenous antioxidants (including GSH) and hence delaying postharvest senescence during storage.

Ascorbic acid and its ascorbate salts are used as anti-browning agents. Ascorbic acid reduces browning of fresh-cut potato (*Solanum tuberosum*) tubers (Tang et al., 2023), fresh-cut lotus roots (Gao et al., 2017), and mung bean sprouts (Sikora and Świeca, 2018). Immersion of basil leaves in a solution of 10 mM ascorbic acid delays the onset of ROS accumulation and chilling injury during low temperature storage (Chotikakham and Janhom, 2025). Ascorbic acid dip solutions of 10 mg L^–1^ promote browning of fresh-cut potato strips during storage at 20 °C, which is not the case for potato strips washed with 5 mg L^–1^ ascorbic acid (Zhou et al., 2021). These authors and their predecessors (Rocculi et al., 2007) postulate the negative ramification of the more concentrated ascorbic acid wash is likely due to the interaction between the exogenous antioxidant and sugars within the tuber that culminate in browning deposits within fresh-cut potatoes. Conversely, Capotorto et al. (2018) found no effect of a 10 mg L^–1^ ascorbic acid wash on the browning of fresh-cut fennel. Apart from browning control, exogenous ascorbic acid applications limit chlorophyll loss and delay the accumulation of ROS in asparagus lettuce (*Lactuca sativa* var. *angustata*) when applied in combination with cysteine and sodium carboxymethyl cellulose (Yu et al., 2023). The disadvantage of using ascorbic acid as a postharvest preservative is its instability in solution. Ascorbic acid is easily degraded in weakly acidic solutions, as well as with light, and non-chilling temperatures (Golubitskii et al., 2007; Yin et al., 2022).

**Figure 3:**
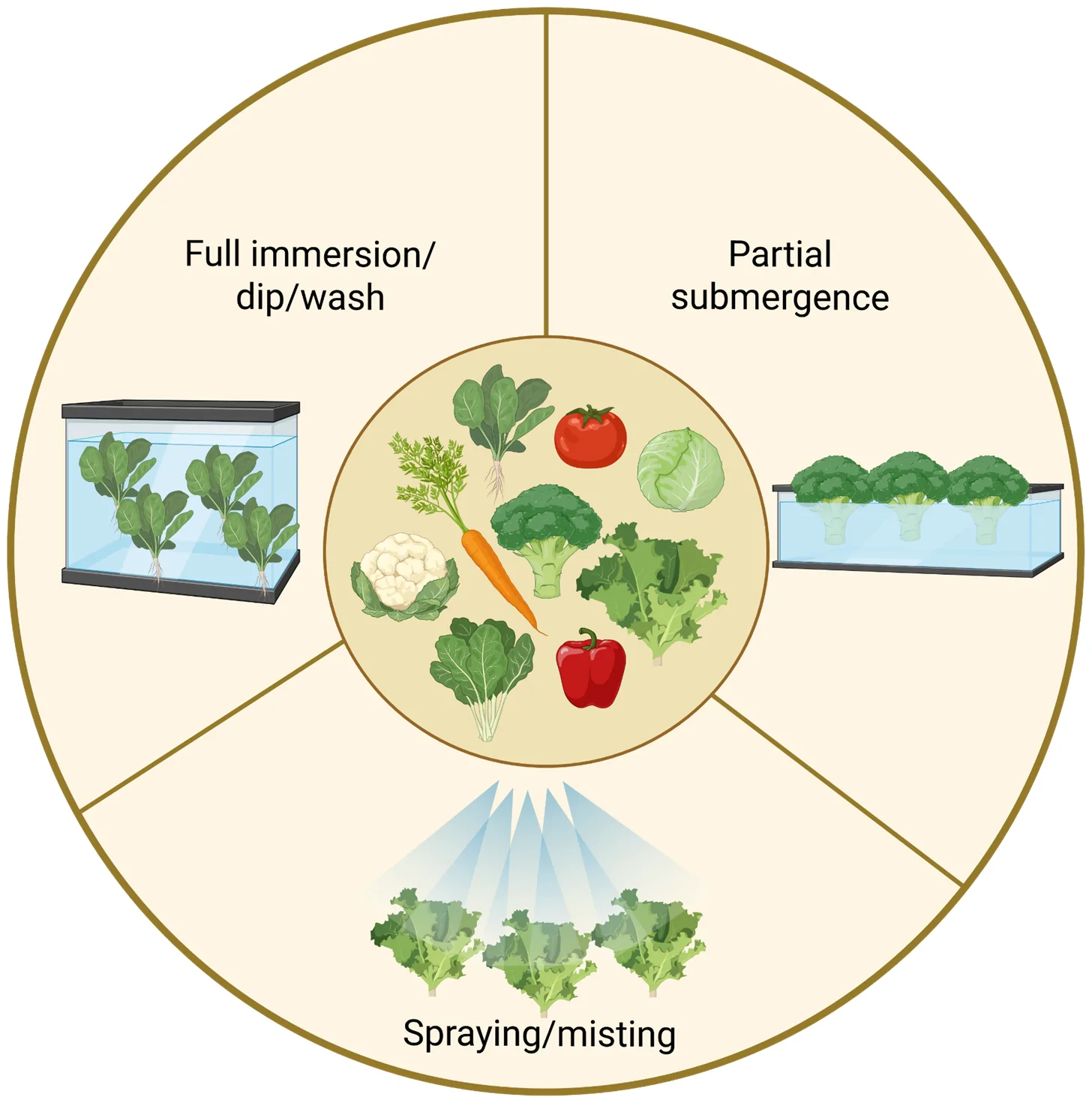
Application strategies of exogenous antioxidants as a strategy to limit senescence and deterioration of vegetables. Vegetables that are amenable to postharvest wetting can be fully immersed in an antioxidant solution for a brief period (e.g. minutes), partially submerged (e.g., minutes to hours) as has been explored for broccoli heads (Yan and Bozzo, 2026) or misted/sprayed prior to storage and/or marketing. Created in BioRender. Bozzo (2026) https://BioRender.com/m68rdv3.

In comparison to ascorbate and ascorbic acid-based applications, GSH and the mushroom-derived antioxidant ergothioneine are generally more stable. In fact, ergothioneine solutions are stable for 32 h under ambient temperatures (Ey et al., 2007). In addition, GSH has a dose-dependent stabilizing effect on ascorbic acid when present at sub-millimolar concentrations (Touitou et al., 1996). Research has established benefits of the pre-storage application of exogenous GSH on the postharvest quality of edible horticulture, including leafy green vegetables. Typically, vegetables are exposed to exogenous antioxidants including GSH through full immersion, partial submergence, or misting (**Figure 3**). Xia (2013) determined that white button mushroom (*Agaricus bisporus*) slices are less prone to browning during hot air-drying following treatment with GSH (0.08% w/v). Yao et al. (2021) found spraying bell peppers with exogenous GSH (0.05% w/v) boosts the endogenous GSH pool by 20%, which was found to be correlated with less superoxide, hydrogen peroxide and chilling injury symptoms relative to untreated peppers. Moreover, the control of ROS in the GSH-treated peppers coincides with increased pools of endogenous ascorbate and greater activities of various ascorbate-GSH recycling pathway enzymes (Yao et al., 2021). GSH applied at concentrations as high as 10 g L^–1^ (i.e., 32.5 mM) limits browning of okra seed pods and lotus root slices (Ali et al., 2023; Li et al., 2023). Green mango (*Mangifera indica*) fruit are also firmer, greener and undergo less fresh weight loss when soaked in 5 mM GSH prior to storage at 20 °C for up to 8 days (Zhou et al., 2023). Similarly, green *Capsicum annuum* pepper fruit immersed in a solution of 10 mM GSH for 40 minutes tend to be greener, firmer and accumulate smaller amounts of hydrogen peroxide and superoxide relative to untreated fruit (Pal et al., 2024). A smaller concentration of 3.25 mM GSH renders deshelled chestnuts firmer and less brown after 20 days at 25 °C relative to untreated chestnuts (Dong et al., 2026). Moreover, a brief 5-minute immersion of broccoli florets in 3.25 mM GSH improves the efficiency of the ascorbate-GSH pathway and limits ROS accumulation during short-term storage at 10 °C (Jiao et al., 2025). Apart from this metabolic benefit, exogenous GSH treatment can limit fermentative metabolism of postharvest horticulture. To this end, less accumulation of ethanol and acetaldehyde and more NAD^+^ are evident in broccoli heads when their stalks were immersed in a solution of 1.3 mM GSH for 3 hours prior to controlled atmosphere storage under elevated CO_2_ and elevated O_2_ partial pressures (Wang et al., 2020). A 30-minute dip in a solution of 0.5 mM GSH limits leaf yellowing and decay of baby wild rocket leaves held at 10 °C for 10 days, a phenomenon associated with little alteration in GSSG levels (Sivakumar and Bozzo, 2024). This same study determined that the potent mushroom antioxidant ergothioneine (i.e., 100 µM) has similar benefits on leaf quality and shelf-life at this temperature. Yan and Bozzo (2026) found that a partial submergence of broccoli heads in a solution of ergothioneine (i.e., 20 µM) for 24 hours limits chlorophyll loss and the buildup of hydrogen peroxide in broccoli florets over a 10-day storage period at 10 °C, which coincides with a more functional ascorbate-GSH pathway relative to broccoli treated with an antioxidant-free solution. Interestingly, this same study established that the partial submergence of broccoli at greater ergothioneine concentrations (e.g., 100 and 500 µM) culminates in more oxidative stress as there is less relatively maintenance of the ascorbate-GSH pathway. Alternatively, the buildup of hydrogen peroxide in broccoli submerged under 100 µM or 500 µM ergothioneine could be due to increased chelation of metals which would limit the metal-dependent conversion of hydrogen peroxide to the hydroxyl radical via the Haber-Weiss cycle (Wang et al., 2024b).

Any antioxidant application strategy should consider whether the commodity of interest can tolerate wetting of its surface. Horticultural commodities that are not amenable to postharvest washing and/or rinsing tend to be susceptible to microbial decay. Typically, commodities that can sustain exposure to postharvest washing are those that contain a cuticle layer on their external surface. Thus, postharvest vegetables that are washed and/or misted in the supply chain would be most suitable for postharvest application of aqueous solutions containing antioxidants. Misting is often used on vegetables within retail display cases as a strategy to limit moisture loss (Brown et al., 2004). Alternative delivery methods that may be promising for the postharvest application of antioxidants to horticulture include fogging or variants thereof (e.g., nano-misting and ultrasonic humidification) (Saenmuang et al., 2012; Perkins et al., 2017). These fogging technologies now have the capacity to produce small droplets that easily evaporate from the surface of the target which should reduce the amount of condensate on the product, and hence spoilage. Water-based fogging together with low temperature storage is known to delay the onset of browning in white asparagus spears (Tirawat et al., 2017). In the aforementioned study fine water droplets were generated from electronic voltages applied to piezoelectric ceramic devices installed in the water tank system that delivered the fog. It is likely that additional delays in postharvest senescence and/or deterioration can be achieved if natural antioxidants are included in washing, misting or fogging solutions that are applied to vegetables in the retail market.

## 4 Conclusion

Distribution of vegetables through the postharvest supply chain is challenged by intermittent and/or prolonged temperature abuse. Thus, investigations focusing on the relationships between vegetable quality and oxidative stress metabolism are pivotal to developing a better understanding of how temperature abuse impacts biochemical responses that induce senescence. Vegetable senescence is intimately associated with alterations in GSH pools and redox status, including the partial to full collapse of the ascorbate-GSH recycling pathway. Conversely, there has been little attention on the relationship between competing biochemical and molecular processes that use GSH as a physiological substrate. It remains to be determined whether an increase in protein *S*-glutathionylation events occur in postharvest vegetables and how these processes impact senescence. Proteomics-based tools would be beneficial in identifying *S*-glutathionylated proteins in vegetables as a function of postharvest storage.

In the absence of *de novo* biosynthesis of GSH, GSH redox status in most vegetables is likely susceptible to catabolic processes associated with one or more of the following enzymes: γ-glutamyl cyclotransferases, GGT, OXP, and LAP. The postharvest accumulation of 5-oxoproline and cysteinylglycine in horticulture implies catabolism of GSH and GSSG. In addition, there is emerging evidence that deaminated GSH is formed from GSH in plants, although the specific GSH transaminase has not yet been elucidated. Recycling of deaminated GSH to GSH is dependent upon a Nit1 ω-amidase, although the role of GSH transamination and recycling has not yet been explored in senescing plants. The restriction of GSH and GSSG catabolism and/or increased recycling of deaminated GSH to GSH are feasible targets to improve GSH redox status in postharvest vegetables. The advent of CRISPR-Cas9 technology provides transferable tools for bioengineering non-model plant species (e.g., arugula) with altered levels of GSH catabolism activities. Germplasm containing gene knockouts or overexpression systems of specific glutathione catabolic steps would provide a mechanism to assess the functional relevance to GSH turnover and plant senescence.
